# High rates of neonatal polycythemia and hyperbilirubinemia during the first phase of COVID-19 pandemic in Italy: a single-center experience

**DOI:** 10.1186/s13052-022-01293-8

**Published:** 2022-06-16

**Authors:** Alice Monzani, Valentino Remorgida, Ivana Rabbone

**Affiliations:** 1 Division of Pediatrics, Department of Health Sciences, Università del Piemonte Orientale, Via Solaroli 17, 28100 Novara, Italy; 2 Division of Obstetrics and Gynecology, Department of Translational Medicine, Università del Piemonte Orientale, Via Solaroli 17, 28100 Novara, Italy

**Keywords:** COVID-19, SARS-CoV-2, Neonates, Newborn, Hyperbilirubinemia, Polycythemia

## Abstract

In our third-level Neonatal Unit in Northern Italy, we recorded a high rate of neonatal hyperbilirubinemia requiring phototherapy in March-November 2020, during the first phase of COVID-19 pandemic, compared to the previous year (198/1348, 14.2%, vs 141/1432, 9.8%, *p* = 0.0004). Supposing it could be the result of neonatal polycythemia, we evaluated capillary hematocrit (Hct) and the rate of hyperbilirubinemia in all newborns ≥36 weeks gestational age born in December 2020. Out of 73 neonates, 37 had Hct ≥65% (50.7%). However, as capillary blood samples may overestimate Hct by 5-15%, even downsizing all values by 15%, Hct was still ≥65% in 9/73 neonates (12.3%), much higher than 0.4-5% prevalence of polycythemia reported in healthy newborns. All those newborns were singleton and healthy, with no clinical signs of hyperviscosity and no underlying factors predisposing to polycythemia. Out of 73 newborns, 13 (17.8%) developed hyperbilirubinemia requiring phototherapy. Their mean Hct value was 66.3 ± 8.2%. Since hyperbilirubinemia is common in the offspring of women with SARS-CoV-2 infection and we recorded increased rates of neonatal hyperbilirubinemia in the first phase of COVID-19 pandemic, it could be hypothesized that even asymptomatic Sars-CoV2 infection during pregnancy might cause placental vascular malperfusion, eliciting polycythemia in the fetus as a compensatory response, that could be the link between COVID-19 in the mothers and hyperbilirubinemia in the newborns.

## Main text

In our Neonatal Unit, a third-level center in Northern Italy, we recorded an increased rate of neonatal hyperbilirubinemia requiring phototherapy in 2020 (March-November), during the first phase of the COVID-19 pandemic, compared to the same time frame in 2019 (198/1348, 14.2%, vs 141/1432, 9.8%, *p* = 0.0004). We hypothesized that it could be the result of neonatal polycythemia, leading to a higher degree of hemolysis, resulting in hyperbilirubinemia. To test this hypothesis, we systematically performed a capillary gas analysis providing hematocrit (Hct) value in all newborns ≥36 weeks gestational age born in December 2020, at the time they underwent newborn screening. In the same month, we recorded the rate of newborns with hyperbilirubinemia requiring phototherapy during hospital stay. Out of 73 neonates born in December, 37 had Hct ≥65% (50.7%). However, as capillary blood samples may overestimate Hct by 5-15%,[1] even downsizing all values by 15%, Hct was still ≥65% in 9/73 neonates (12.3%), much higher than 0.4-5% prevalence of polycythemia reported in healthy newborns in the literature [1] and 0.3% found in our Neonatal Unit in March-November 2019. All those newborns were singleton and healthy, with no clinical signs of hyperviscosity. No underlying factors predisposing to polycythemia were found in their maternal or perinatal history. None was born to mothers with gestational diabetes, preeclampsia, or smoking in pregnancy. Their mean gestational age was 39 weeks (range 38-41). In 8/9 the birth weight was appropriate for gestational age (mean weight 3300 g, range 2880-3970 g), one was small for gestational age, and no one was large for gestational age. The percentage of weight loss from birth was similar in newborns with or without polycythemia (7.4 ± 2.5% vs. 8 ± 1.7%, NS). Out of 73 newborns, 13 (17.8%) developed hyperbilirubinemia requiring phototherapy. Their mean Hct value was 66.3 ± 8.2%.

Hyperbilirubinemia is one of the neonatal outcomes commonly reported in the offspring of women with SARS-CoV-2 infection [2–6]. Since the increased rate of neonatal jaundice in our unit was recorded during the first phase of COVID-19 pandemic, it could be hypothesized that neonatal hyperbilirubinemia may be an indirect sign of Sars-CoV2 infection in pregnant women, even if not recognized because asymptomatic. It may be supposed that even asymptomatic Sars-CoV2 infection during pregnancy might result in placental vascular malperfusion [7, 8], eliciting polycythemia in the fetus as a compensatory response, finally resulting in a greater probability of developing hyperbilirubinemia. Indeed, we observed an increased rate of neonatal hyperbilirubinemia following a period of high rates of positive testing for COVID-19 in Northern Italy.

The main limitation of this study was not having performed a Sars-CoV2 test during pregnancy in the mothers of newborns with hyperbilirubinemia, because in the first phase of the pandemic the frequency of swab test was very low in asymptomatic subjects.

In conclusion, a high prevalence of polycythemia and hyperbilirubinemia in a cohort of healthy neonates born during the first phase of COVID-19 pandemic was observed. Any possible change in cord clamping practices was excluded. Placental malperfusion even in women with asymptomatic Sars-CoV2 infection, resulting in increased fetal erythropoiesis, could explain these findings. Further systematic observational studies are needed to confirm this hypothesis.

## Data Availability

All data generated or analysed during this study are included in this published article.

## Abbreviation

Hct: Hematocrit
